# Transcriptomic signatures and immune microenvironment of acute rejection after heart transplantation: an integrated bioinformatics analysis

**DOI:** 10.3389/fcvm.2026.1796145

**Published:** 2026-05-25

**Authors:** Wenjun Zhou, Langjing Huang, Wenwen Tang

**Affiliations:** 1 Department of Anesthesiology, The Sixth Hospital of Wuhan, Affiliated Hospital of Jianghan University, Wuhan, China; 2 Department of Cardiovascular Medicine, Changsha Economic Development Zone Hospital, Changsha, China; 3 Department of Cardiology, Wuhan No.9 Hospital, Wuhan, China

**Keywords:** acute rejection, biomarker, endomyocardial biopsy, heart transplantation, immune microenvironment, interferon signaling, LASSO, transcriptome

## Abstract

**Background:**

Histologic assessment of endomyocardial biopsy (EMB) remains the standard for diagnosing acute cardiac allograft rejection, yet molecular profiling may provide complementary quantitative insights.

**Methods:**

Microarray data (GSE2596; GPL1053) were obtained from the Gene Expression Omnibus. Acute rejection (R, *n* = 16) and non-rejection (N, *n* = 27) samples were analyzed after excluding infection-related groups. Probes were mapped to gene symbols and summarized per gene. Differential expression was assessed using Welch's *t*-test with Benjamini–Hochberg false discovery rate control. Reactome over-representation analysis was performed for significant genes. Immune cell scores were estimated using MCP-counter markers. An L1-penalized logistic regression model was evaluated by 5-fold cross-validation. Experimental validation was performed by quantitative PCR in a cervical heterotopic cardiac xenotransplantation model (BALB/c to C57), including normal control and sham groups.

**Results:**

Among 3,968 genes, 1,032 were differentially expressed (FDR<0.05), including 135 with |log2FC|>1. Upregulated genes included HLA-DMA, DEF6, TRB@, and CD74. Enrichment analysis highlighted interferon signaling, T-cell receptor signaling, chemokine pathways, and antigen presentation. Immune scoring indicated increased monocytic, B-lineage, and T/cytotoxic lymphocyte signals in rejection. The diagnostic model achieved strong discrimination (5-fold AUC=0.993). qPCR confirmed coordinated upregulation of interferon-related (Ifng, Stat1), chemokine (Cxcl10), and cytotoxic (Prf1) genes in transplanted grafts.

**Conclusions:**

Acute rejection EMB transcriptomes demonstrate coordinated interferon-driven immune activation. A compact gene signature shows strong internal diagnostic performance, supported by experimental validation, warranting external confirmation.

## Introduction

In addition to the substantial advancements that have been made in immunosuppressive regimens and perioperative *management*, Acute cellular and antibody-mediated rejection is still one of the most significant early complications following heart transplantation (1). Acute rejection can lead to decreased graft function, repeated Injury to the Allograft, and it may also accelerate the development of Chronic Allograft Vasculopathy, all of which can impact long-term survival (2). Therefore, prompt identification and risk stratification remain an integral part of post-transplant care; meanwhile, clinical manifestations of both rejection and infection remain non-specific and may overlap due to either drug toxicity or ischemia-reperfusion injury (3).

Endomyocardial biopsy (EMB) with histologic evaluation, using standardized grading systems, is the standard clinical reference for the diagnosis of rejection in heart transplant patients (4). However, EMB has limitations due to focal disease and sampling variability, and the interpretation of EMB biopsy specimens can be influenced by inter-observer variation. Importantly, graft molecular immune activity may occur prior to the observable appearance of overt histopathologic changes, indicating that reliance on histology alone may delay detection of rejection in some patients (5). This has driven the movement to complement the conventional histological grades with objective molecular measures of intragraft immune activity. Transcriptomic profiling of EMB provides a quantitative framework that depicts the concurrent immune, inflammatory, and stromal changes that occur in the Allograft during rejection. Prior studies in transplantation medicine have demonstrated that gene expression patterns can be used to distinguish between the various phenotypes of rejection and potentially provide insights into mechanisms of T-cell activation, interferon-signaling, antigen presentation, and complement-mediated injury (6). However, Variability in reported signatures exists between cohorts and research platforms. In addition, models must be robust, interpretable, and subject to transparent validation; therefore, the need exists for such models for datasets containing clinical labels with good annotations.

This study aimed to integrate bioinformatics analysis of a publicly available EMB microarray dataset to analyze gene and pathway characteristics of acute rejection. Specifically, the study aimed to (i) determine the gene expression differential patterns for acute rejection vs. non-rejection, (ii) identify enriched Reactome pathways to contextualize the biological processes associated with acute rejection. Additionally, (iii) we estimated the immune and stromal population signals using marker-based scoring to approximate the graft microenvironment; (iv) develop an internally validated sparse gene signature to facilitate discrimination of acute rejection. Together, this Information provides a structured map of altered transcriptomic patterns during acute rejection along with potential candidate biomarkers and Pathways to be utilized in future translational validation and clinical decision support.

## Materials and methods

### Data source and sample selection

Expression profiles were obtained from GEO series GSE2596. The platform was GPL1053 (*N*IAID-Hsab), a spotted oligonucleotide microarray. Sample group annotations were extracted from the series matrix sample titles. We restricted the primary analysis to samples labeled as acute rejection (R) and non-rejection/non-Chagas (N); samples labeled as pre-rejection, pre-Chagas, Chagas reactivation, toxoplasma myocarditis, and other diagnoses were excluded to reduce etiologic heterogeneity. Raw and processed expression data and accompanying metadata for GSE2596 on platform GPL1053 were retrieved from the NCBI Gene Expression Omnibus. The analysis used the GSE2596 series matrix file together with the corresponding GPL1053 platform annotation file. Accession identifiers, the download date, and file names with checksums should be logged in the analysis repository to enable exact reproduction.

### Microarray preprocessing and normalization

The GSE2596 series matrix file provides processed log2 expression ratios for the GPL1053 two color spotted oligonucleotide microarray. Raw image level intensity files were not available in GEO, so background correction and within array normalization could not be re applied. Accordingly, downstream preprocessing focused on probe annotation, gene level summarization, filtering based on missingness, and z scoring where required for downstream analyses.

### Probe annotation and gene-level summarization

Gene identifiers were harmonized using the GPL1053 platform annotation file. Certain probe annotations such as ‘TRB@’ correspond to the T-cell receptor beta locus rather than a single HGNC gene symbol. To ensure transparency and reproducibility, we retained the original platform annotation while documenting the corresponding probe identifiers and mapping logic. Detailed probe-to-gene mapping information for signature genes is provided in Supplementary Table S1. Probe identifiers (ID_REF) were mapped to gene symbols using the GPL1053 platform annotation (family SOFT file). For genes represented by multiple probes, expression values were summarized by the median across probes. Genes with fewer than 80% non-missing values across retained samples were removed; remaining missing values were imputed by the gene-wise mean (7). Missingness summary and justification. After probe to gene summarization and filtering for at least 34 of 43 samples with non missing values (approximately 79% completeness), 3,968 genes were retained. Across this retained matrix, 6.6% of gene by sample entries were missing (11,231 of 170,624). Per gene, the median number of missing values was 2 (75th percentile 5; 95th percentile 8), with a maximum of 9 by design. Per sample, missingness ranged from 0.2% to 22.9% of genes (median 4.2%). Gene wise mean imputation was used only for steps requiring complete matrices (PCA, correlation heatmaps, and model fitting). Differential expression and pathway analyses were repeated without imputation (complete case genes) and produced concordant strict DEG and pathway results (Supplementary Table S1). As an additional sensitivity check, PCA and sample correlation structure were recomputed using complete case genes without imputation.

### Quality control and exploratory analysis

Principal component analysis (PCA) was performed using gene-level expression values (8). Sample-to-sample Pearson correlation was computed using the top 1,000 most variable genes and visualized as a clustered correlation heatmap.

### Differential expression analysis

Differential expression between R and N was evaluated at the gene level using Welch's two-sample t-test (9). Log2 fold change was defined as the difference in mean expression (R minus N). Multiple testing was controlled using the Benjamini–Hochberg FDR. Genes with FDR<0.05 were considered significant; for enrichment analyses, we additionally required |log2FC|>1. Effect size reporting and threshold justification. Differential expression results are reported together with effect sizes (log2 fold change) and statistical uncertainty controlled by Benjamini–Hochberg FDR. The volcano plot thresholds (FDR < 0.05 and |log2FC| > 1) were selected to balance statistical significance and biological relevance and are commonly used in transcriptomic studies. Confidence intervals for model discrimination metrics are reported using bootstrap resampling. As robustness checks, differential expression results were compared across alternative preprocessing and inference choices, including median versus mean probe to gene summarization, alternative missingness thresholds, analyses without imputation, and a nonparametric Mann Whitney U test.

### Pathway enrichment

Reactome over-representation analysis (ORA) was conducted using Fisher's exact test to assess enrichment of significantly changed genes among curated Reactome pathways (gene set size 10–500) (10). FDR correction was applied across tested pathways. Threshold sensitivity. Because over-representation analysis (ORA) relies on predefined DEG thresholds, enrichment results were interpreted cautiously and in the context of the overall DEG distribution. Threshold-free complementary approaches such as gene set enrichment analysis (GSEA) may provide additional insight in future analyses and are noted here to clarify sensitivity to DEG cutoff selection.

### Immune and stromal population scoring

Immune and stromal signals were estimated using MCP-counter marker gene sets. Gene expression was z-scored across samples, and each population score was computed as the mean z-score of marker genes present in the dataset. Group differences were assessed using Welch's *t*-test (11). Marker coverage assessment for MCP-counter. Because MCP-counter scores depend on the availability of marker genes on the microarray platform, we examined marker coverage for each immune cell population using the GPL1053 annotation. Only marker genes present on the platform were retained for score calculation, and the detected marker sets for each cell type were reported. Several MCP-counter populations could not be computed because corresponding marker probes were absent on the array platform. Therefore the resulting cell scores should be interpreted as approximate indicators of immune activity rather than precise quantitative estimates of immune infiltration.

### Diagnostic signature modeling

To derive a compact diagnostic model, we selected the top 30 differentially expressed genes (FDR<0.05; ranked by absolute log2FC) and fitted an L1-penalized logistic regression model (12). Model performance was evaluated using 5-fold stratified cross-validation with out-of-fold predicted probabilities; discrimination was summarized by ROC-AUC. To address potential information leakage, we performed a sensitivity analysis using nested cross validation in which gene ranking and selection were repeated within each training fold, and scaling and model fitting were applied only to training data via a scikit learn Pipeline; performance was computed from out of fold predictions. Recipient identifiers were not available in the public GEO metadata, so we could not determine whether multiple biopsies were obtained from the same transplant recipient. Cross validation was therefore performed at the biopsy level and may be optimistic if repeated measures are present; we present these results as internal, exploratory estimates and emphasize the need for patient level validation. We quantified uncertainty by nonparametric bootstrap resampling of out of fold predictions to obtain 95% confidence intervals for ROC AUC and PR AUC, and evaluated calibration using Brier score and a reliability curve.

### Cervical heterotopic cardiac xenotransplantation model and study groups

A cervical heterotopic cardiac xenotransplantation model was established using BALB/c mice as donors and C57 mice as recipients. Animals were allocated to three groups (*n* = 6 per group). The normal control group (CON) received no surgical manipulation. The sham group underwent anesthesia and right-sided neck exploration followed by ligation of the recipient right common carotid artery and right jugular vein without graft implantation. The xenotransplantation group (Xeno-Tx) received BALB/c-to-C57 cervical heterotopic cardiac xenografts with establishment of graft perfusion. Samples were collected at the predefined time point after surgery or transplantation. The animal experiments presented in this manuscript were approved by the Ethics Committee of Wuhan No. 9 Hospital (approval No. 2025061213).

### RNA extraction, reverse transcription, and quantitative PCR

Total RNA was extracted from collected specimens using a standardized protocol and quantified prior to downstream processing. Complementary DNA was synthesized from total RNA using reverse transcription according to the manufacturer's instructions. Quantitative PCR was performed using gene-specific primers for interferon gamma (Ifng), tumor necrosis factor alpha (Tnf), perforin 1 (Prf1), granzyme B (Gzmb), Cxcl10, and Stat1. Reactions were run with technical replication and included no-template controls. Primer sequences are provided in the Supplementary Materials.

### Software and computational environment

All analyses were conducted in Python 3 using a reproducible script and notebook workflow. Data import, reshaping, and quality control were performed with pandas and NumPy. Statistical analyses, including group comparisons and correlation analyses, were performed with SciPy, and multiple testing correction using the Benjamini Hochberg false discovery rate was implemented with statsmodels. Dimensionality reduction with principal component analysis, feature selection and sparse modeling using L1 regularized logistic regression, and internal validation using cross validation and performance metrics such as the receiver operating characteristic area under the curve were performed with scikit learn. All figures were generated with matplotlib. Where applicable, random seeds were fixed to improve reproducibility. GEO files for GSE2596 and GPL1053 were downloaded from NCBI GEO on 2 November 2025, and accession identifiers, download dates, file names, and checksums were recorded in the analysis repository. Analyses were executed in Python version 3.10 or later with key packages including pandas 2.x, NumPy 1.x, SciPy 1.x, statsmodels 0.14 or later, scikit learn 1.x, and matplotlib 3.x. Full code, parameter settings, and package versions should be provided as a supplementary script or notebook to support exact reproduction. Group comparisons for qPCR data were performed on −*Δ*Ct values using two-sided tests as appropriate, with multiple testing controlled by the Benjamini–Hochberg procedure when evaluating multiple genes.To facilitate full reproducibility of the computational workflow, the complete analysis code, notebooks, software versions, and parameter settings (including random seeds, logistic regression solver, regularization strength selection strategy, and scaling procedures) will be deposited in a public repository such as GitHub or Zenodo. The repository will also record dataset accessions, preprocessing scripts, and figure generation code to allow exact replication of the analyses.

## Results

### Study workflow and biopsy group composition in GSE2596

**Figure 1:**
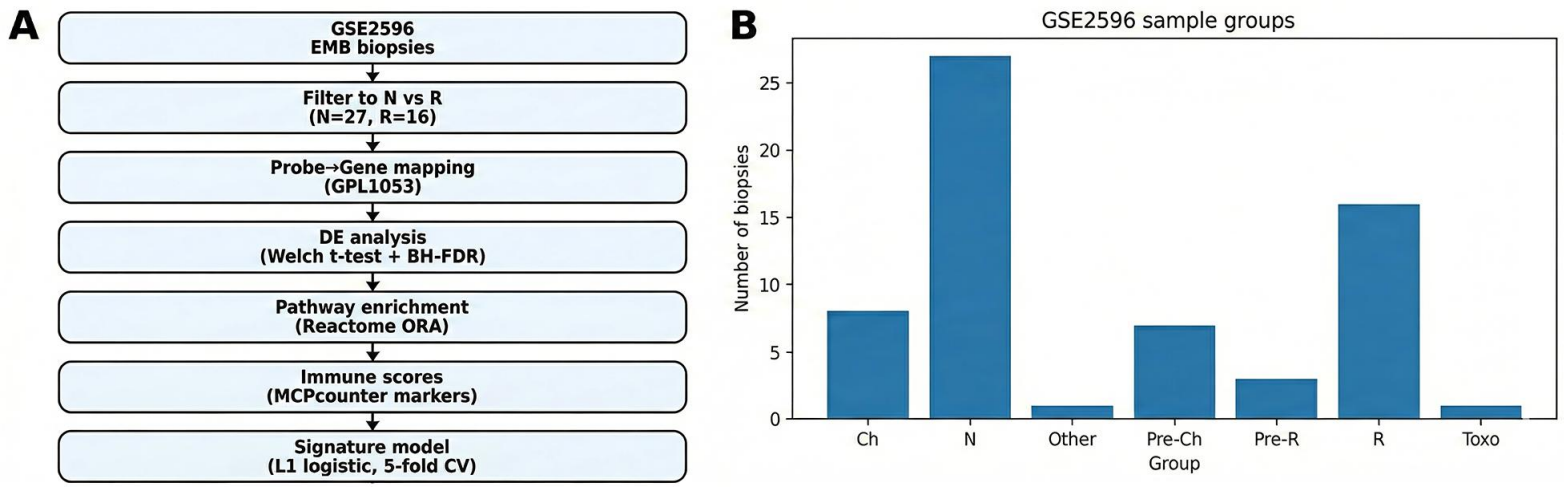
Study workflow and sample groups. *N* = non-rejection, *R* = acute rejection. After exclusions, the primary analysis included *N* = 27 and *R* = 16 EMB samples. Group sizes: *N* = 27, *R* = 16. No statistical comparison was performed in this descriptive overview figure. **(A)** Overview of the analysis pipeline applied to the GSE2596 endomyocardial biopsy dataset. **(B)** Distribution of the original cohort labels in GSE2596. The main analyses focus on N and R.

As summarized in the analysis workflow (Figure 1A), we analyzed endomyocardial biopsy (EMB) transcriptomes from the GSE2596 dataset and focused subsequent differential and downstream analyses on the comparison between non rejection (N) and acute rejection (R) samples. After filtering to the primary contrast, 43 biopsies were retained for the core analyses (*N* = 27; *R* = 16), followed by probe to gene mapping using the GPL1053 platform annotation, gene level differential expression testing (Welch's *t* test with Benjamini Hochberg false discovery rate correction), Reactome over representation pathway analysis, and immune and stromal signal estimation using MCP counter marker scores, culminating in development of an L1 regularized logistic regression signature model with cross validation (Figure 1A).

The original dataset contained multiple clinical groups beyond the primary N and R comparison, including Ch, Pre Ch, Pre R, Toxo, and Other categories (Figure 1B). This group structure supported an initial overview of cohort composition while motivating the focused N versus R analysis to address the principal biological question of acute rejection associated transcriptional changes.

### Dataset characteristics

**Figure 2:**
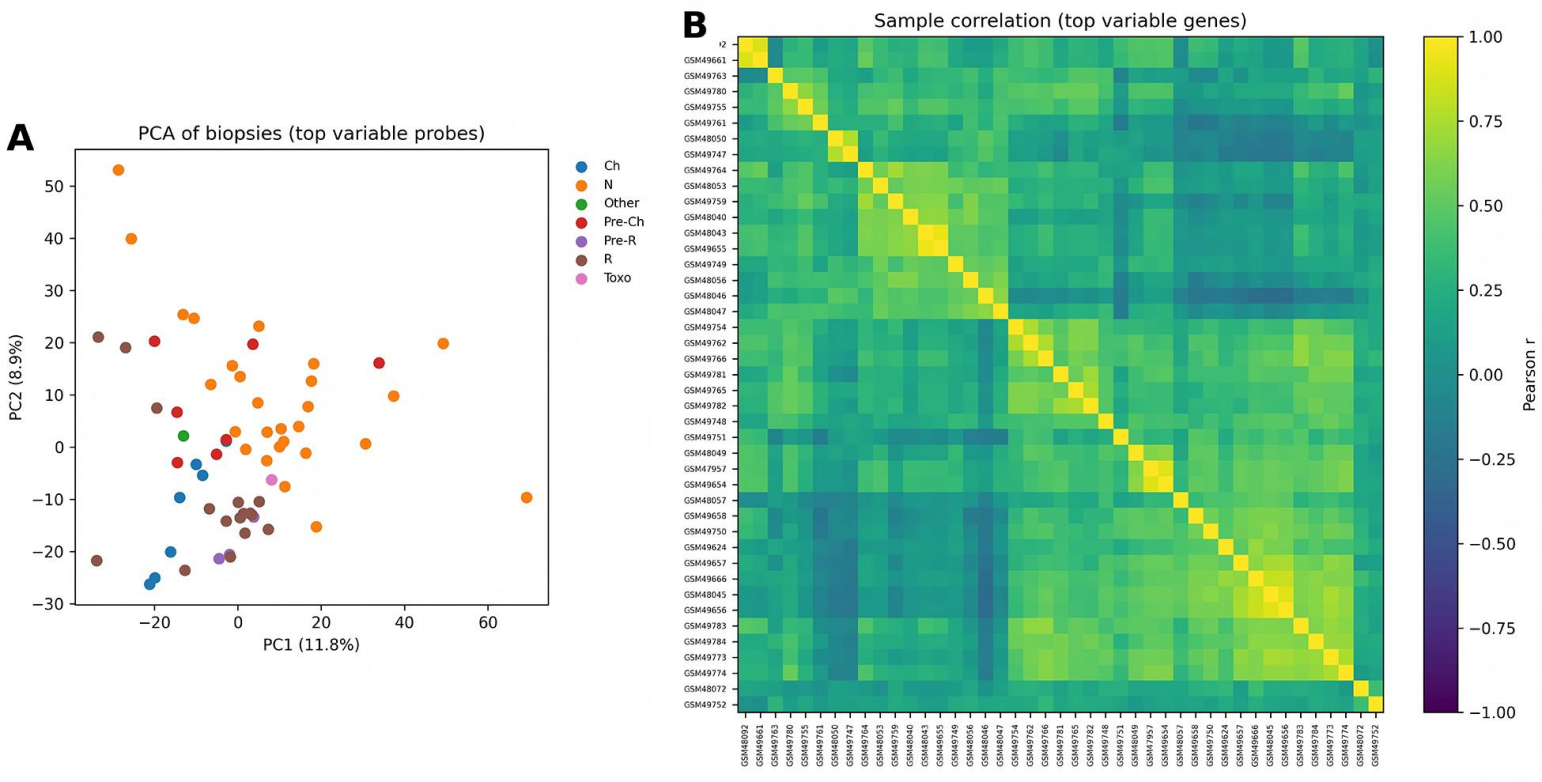
Global structure of expression data across biopsy samples. PCA was performed on gene-level expression after probe-to-gene summarization; samples are colored by clinical label. Correlations are Pearson's r; dendrogram indicates hierarchical clustering. Group sizes: *N* = 27, *R* = 16. PCA performed on gene-level expression values; correlations computed using Pearson's r. No multiple-testing correction was required for this exploratory visualization. **(A)** Principal component analysis based on the top variable probes, with points colored by the original cohort label. Axes show the percent variance explained by PC1 and PC2. **(B)** Sample to sample Pearson correlation heatmap computed from the same set of top variable probes, with hierarchical clustering of samples.

The GSE2596 dataset comprised endomyocardial biopsy transcriptomes spanning multiple clinical categories (Ch, N, Other, Pre-Ch, Pre-R, R, and Toxo). Principal component analysis using the most variable probes showed that samples exhibited structured heterogeneity, with a tendency for biopsies to distribute according to clinical labels and with R and N showing partial separation along the leading components (Figure 2A). Consistently, a sample–sample correlation heatmap based on highly variable genes revealed blocks of higher within-group similarity, indicating that transcriptomic profiles were more similar among samples sharing the same clinical annotation (Figure 2B). For downstream differential expression and pathway analyses, we focused on the primary comparison between non-rejection and acute rejection biopsies (*N* = 27; *R* = 16). Global structure results were similar in a complete case sensitivity analysis without imputation (Supplementary Figure S1).

### Differential expression landscape

**Figure 3:**
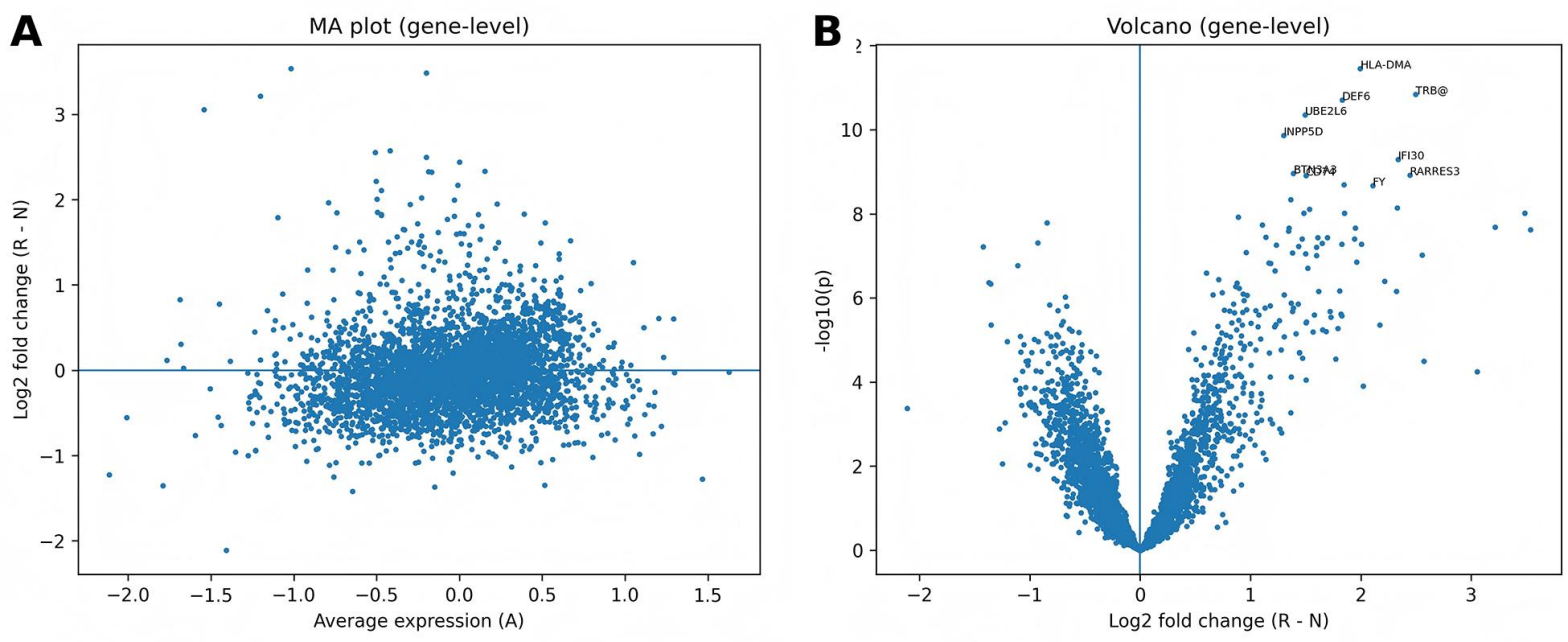
Differential expression between acute rejection and non rejection. Differential expression used Welch's *t*-test with Benjamini–Hochberg FDR control; log2FC is mean(R)−mean(N). Thresholds used for labeling/significance are indicated in the plot. Group sizes: *N* = 27, *R* = 16. Differential expression tested using Welch's two-sample t-test with Benjamini–Hochberg FDR correction across genes. **(A)** MA plot at the gene level for the R versus N comparison. The *x* axis shows average expression and the *y* axis shows log2 fold change (R minus N). **(B)** Volcano plot showing log2 fold change (R minus N) versus minus log10(FDR). Selected top ranked genes are labeled.

**Figure 4:**
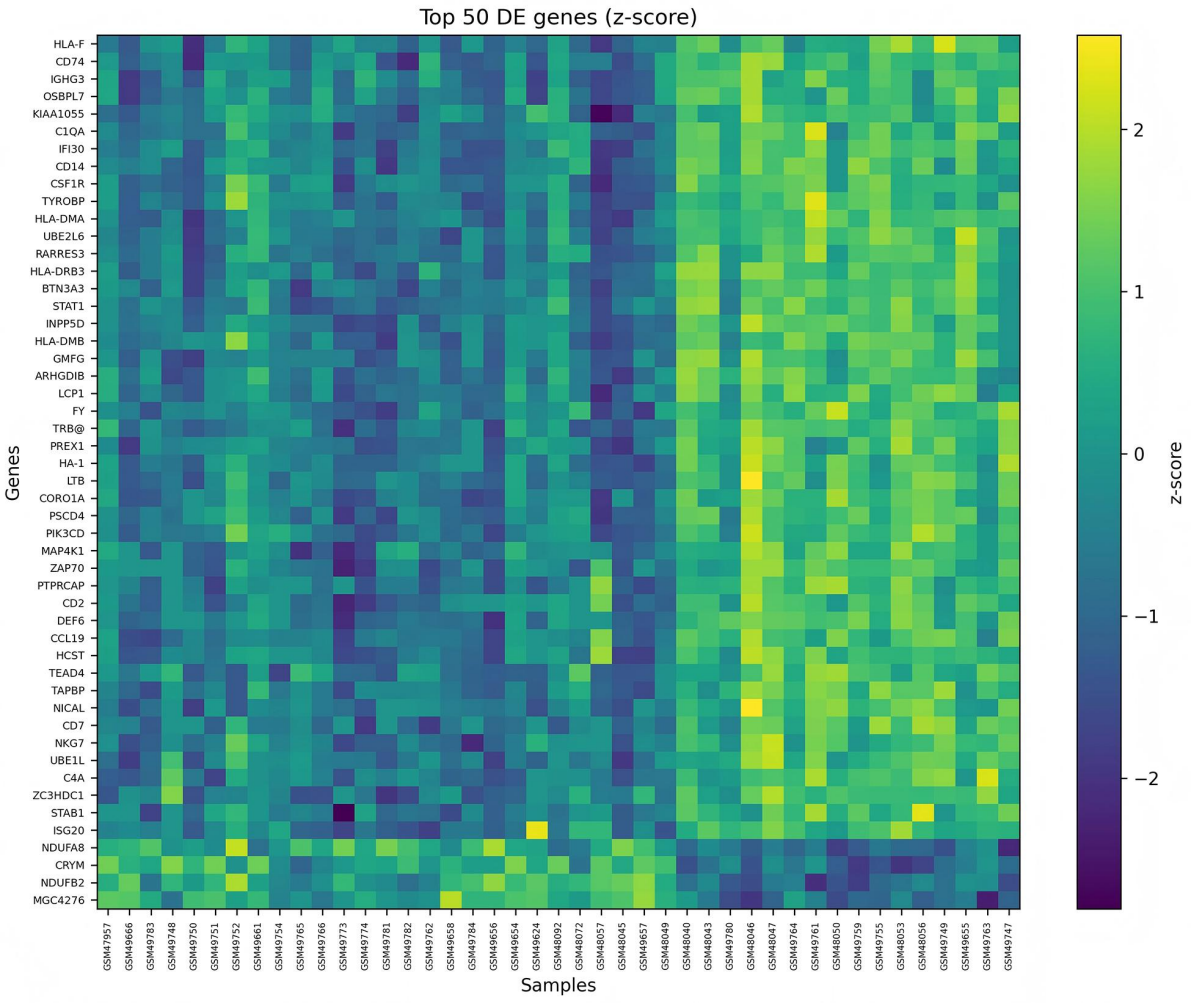
Heatmap of the top 50 differentially expressed genes. Heatmap shows row-wise z-scores; columns are annotated by phenotype (N vs. R) and ordered by group; clustering was performed using hierarchical clustering with an appropriate distance metric. Group sizes: *N* = 27, *R* = 16. Heatmap visualization only; statistical testing for differential expression was performed as described in the Methods using Welch's t-test with FDR correction. Row wise z scored expression is shown for the 50 genes with the smallest FDR in the R versus N comparison. Columns represent samples (ordered by group) and rows are clustered by hierarchical clustering.

From a total of 3,968 annotated genes that survived quality filtering, 1,032 were found to be differentially expressed between acute rejection and non-rejection biopsies at a False Discovery Rate (FDR) of less than 0.05. A more stringent threshold of FDR < 0.05 and a |log2FC| > 1 identified 135 differentially expressed genes in total, of which 109 were more highly expressed in the setting of acute rejection, whereas only 26 had a lower level of expression in the context of acute rejection. Figures 3A,B shows the statistical significance and magnitude of differential expression between groups using the MA and Volcano plots, respectively. Some of the genes that were most significantly increased in expression during an episode of acute rejection were genes involved in antigen presentation and immune activation such as HLA-DMA, CD74, IFI30, DEF6, TRB@, UBE2L6, INPP5D (Figure 3B). The heatmap created using the top fifty differentially expressed genes demonstrates similar patterns of expression across the cohort, where many of the transcripts associated with immune activity were expressed at higher levels during an episode of acute rejection than they were during non-rejection biopsies (Figure 4). Sensitivity analyses using alternative summarization, missingness thresholds, and moderated or nonparametric tests yielded highly concordant DEG lists (Supplementary Table S2).

### Pathway enrichment highlights interferon and adaptive immune signaling

**Figure 5:**
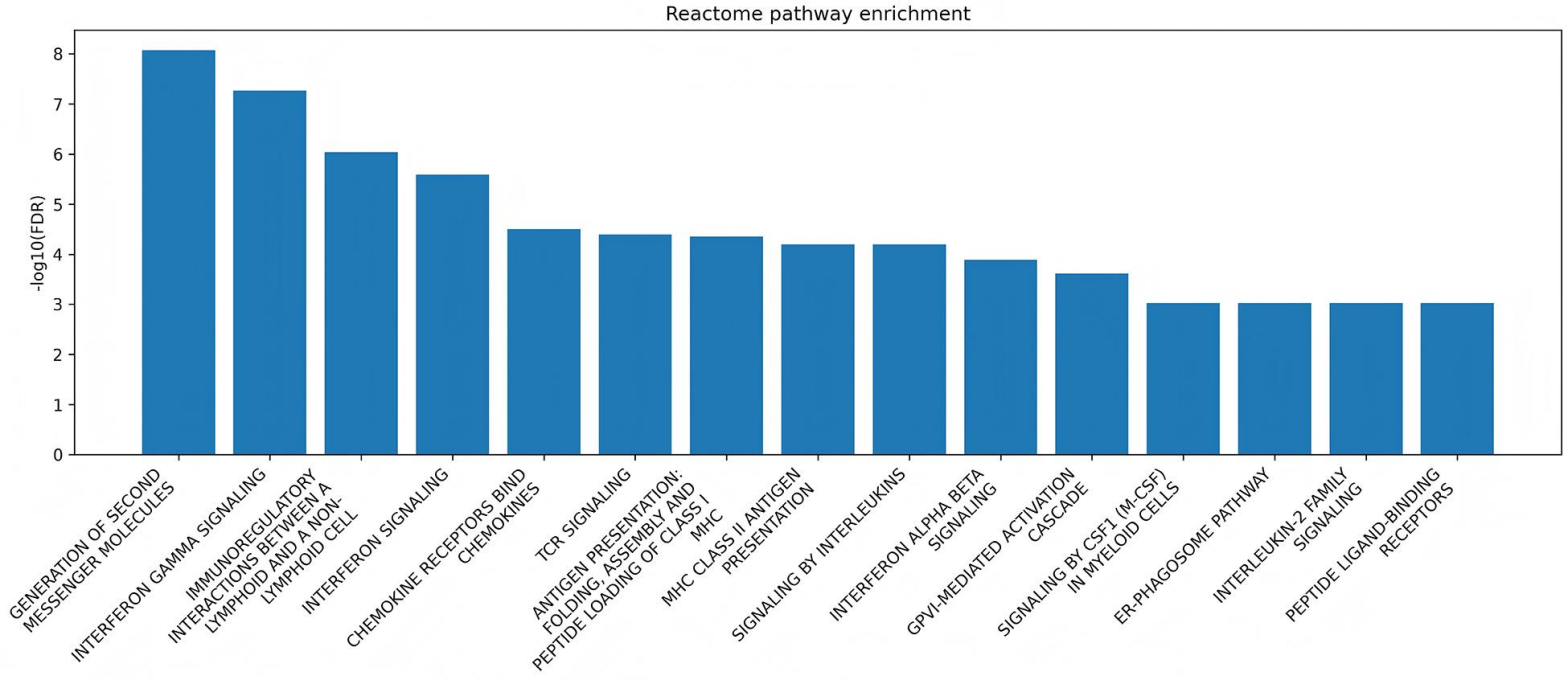
Reactome pathway enrichment of rejection associated genes. Input gene list required FDR<0.05 and |log2FC|>1; enrichment used Fisher's exact test with FDR correction across pathways; only pathways with gene set size 10–500 were tested. Enrichment statistics derived from Fisher's exact test with Benjamini–Hochberg FDR correction across tested pathways.Bar plot of the top enriched Reactome pathways from over representation analysis using genes meeting FDR < 0.05 and absolute log2 fold change > 1. Bar height represents minus log10(FDR).

Reactome over representation analysis was conducted on the genes that had an FDR value less than 0.05 with an absolute log2FC value greater than 1. The majority of enriched pathways associated with the genes were related to immune function. The primary immune pathways included the generation of second messenger molecules; Interferon gamma signaling; Interferon signaling; Immunoregulatory interactions between lymphoid and non lymphoid cells; Chemokine receptors that bind chemokines; T Cell Receptor Signaling; and antigen presentation Pathway with MHC class I and MHC class II; as illustrated in Figure 5.

### MCP counter scores show increased immune infiltration in rejection

**Figure 6:**
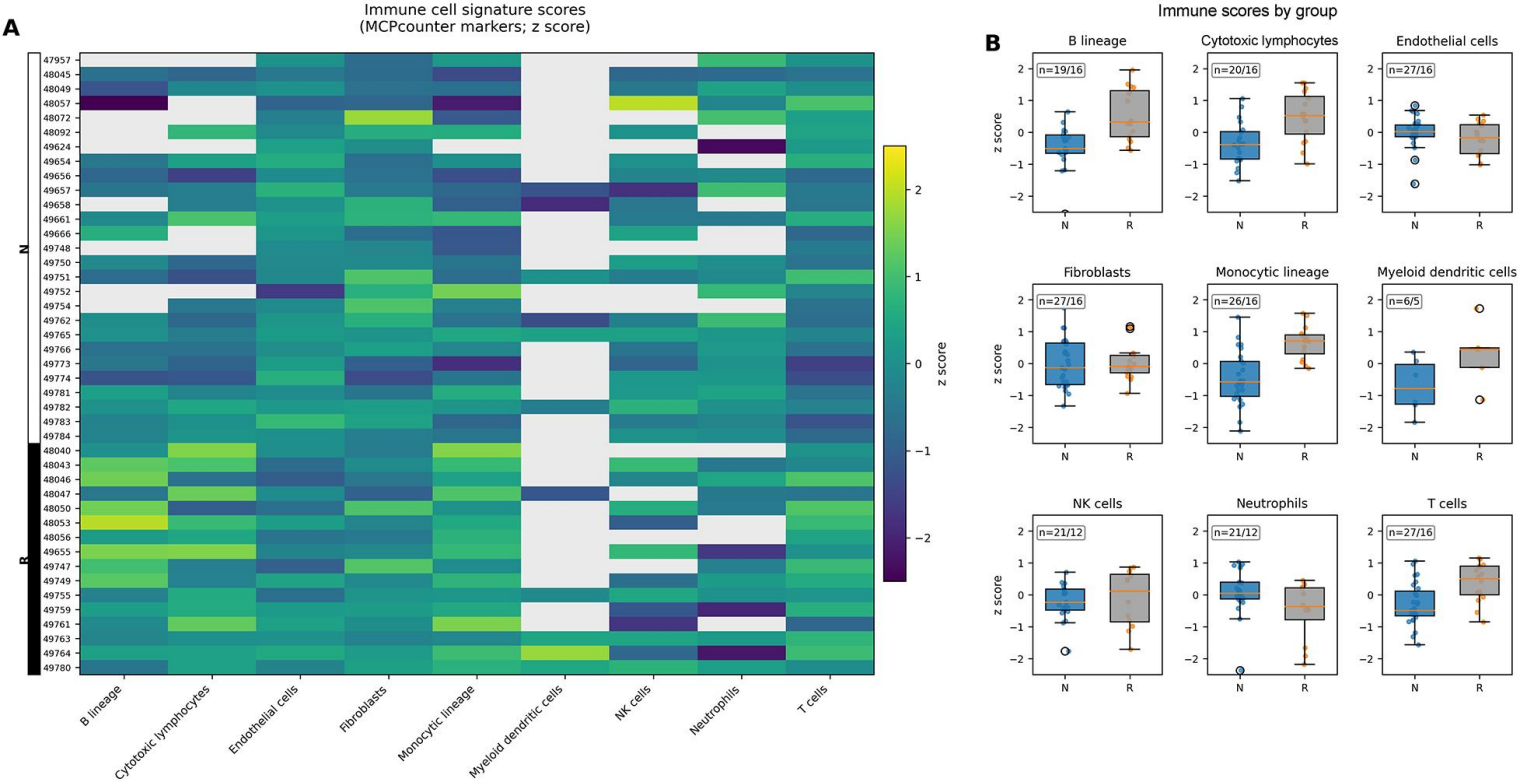
Immune and stromal population signals estimated by MCPcounter. Population scores are the mean z-score of available MCP-counter marker genes per population; grey indicates missing scores due to absent marker probes. Boxplots show median and IQR; *p*-values from Welch's *t*-test. Group sizes: *N* = 27, *R* = 16 (numbers per boxplot correspond to samples with available marker coverage). Group comparisons performed using Welch's t-test without additional multiple-testing correction due to exploratory cell-score analysis. **(A)** Heatmap of MCPcounter population scores across biopsies (mean z score of marker genes). Columns represent immune or stromal populations and rows represent samples ordered by phenotype (N and R). Grey indicates missing scores when required marker probes were not available. **(B)** Boxplots of MCPcounter population scores comparing N versus R. Points show individual samples. Per panel n indicates the number of biopsies with non missing scores in N and R.

The heatmap overview of MCP-counter immune cell scores (Figure 6A) showed a general upward shift of several immune-related signals in rejection biopsies compared with non-rejection samples. In particular, signals corresponding to cytotoxic lymphocytes, B lineage cells, monocytic lineage cells, and T cells appeared more prominent in the rejection group, suggesting increased immune activation within the graft microenvironment. Consistent with the heatmap patterns, group-wise comparisons of MCP-counter scores (Figure 6B) demonstrated significantly higher immune signature scores in rejection samples, most notably for monocytic lineage, B lineage, and T cells (Monocytic lineage *Δ* = 1.13, *p* = 3.13 × 10⁻⁶; B lineage *Δ* = 1.05, *p* = 3.52 × 10⁻⁴; T cells *Δ* = 0.67, *p* = 1.60 × 10⁻³). These findings are consistent with the pathway enrichment results highlighting interferon signaling, T-cell receptor signaling, chemokine signaling, and antigen presentation pathways.

### Sparse eight-gene diagnostic signature distinguishes rejection from non-rejection

**Figure 7:**
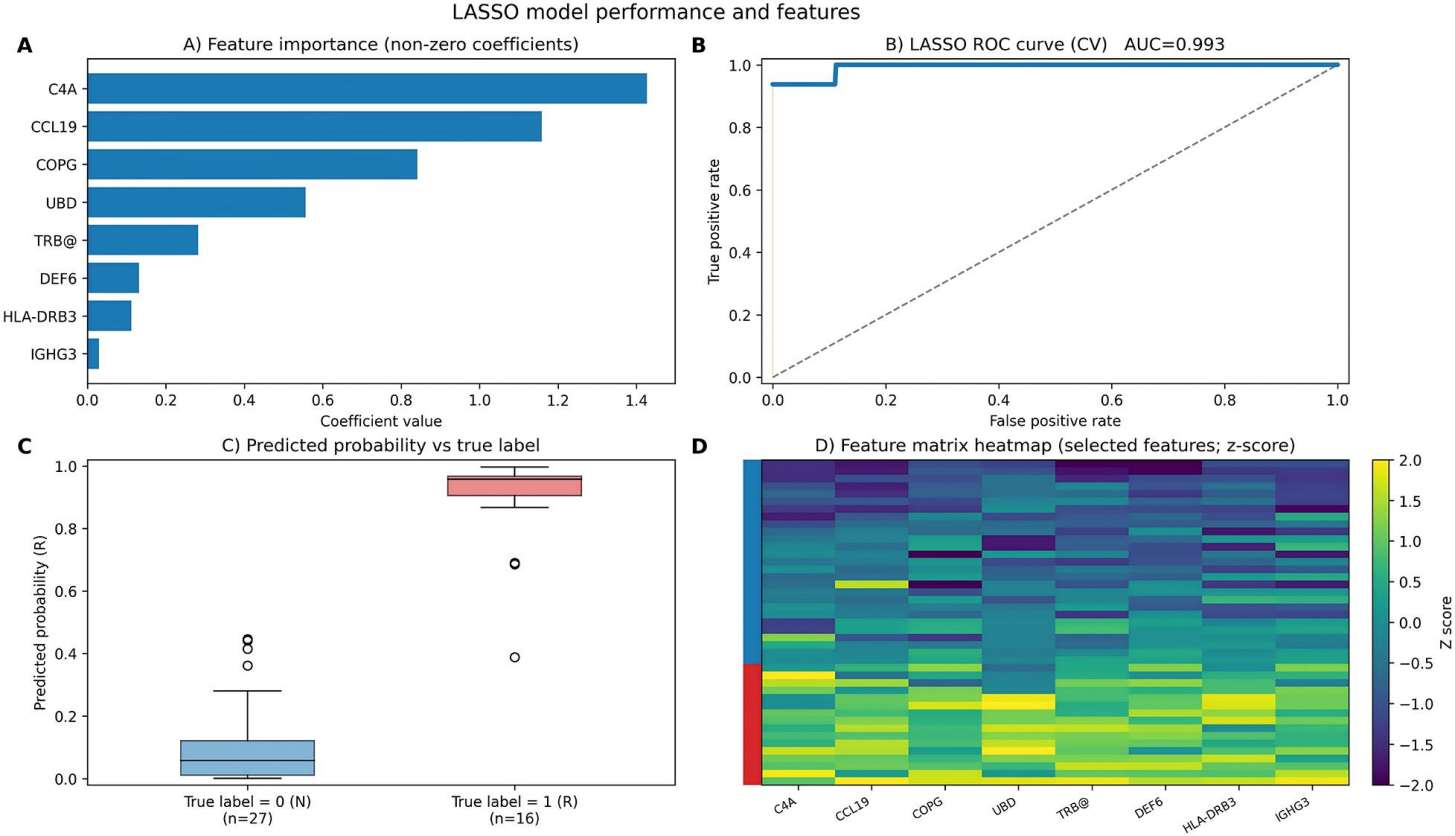
Sparse diagnostic model for acute rejection. L1 penalized logistic regression was trained on the top 30 DE genes (ranked by absolute log2FC) and evaluated by 5 fold stratified cross validation; ROC AUC was computed from out of fold predictions. As a leakage control sensitivity analysis, the full pipeline with within fold feature selection was evaluated using nested cross validation, with ROC and PR curves shown in Supplementary Figure S2 (ROC AUC 0.956; PR AUC 0.943). Calibration of the nested cross validation predictions was assessed by Brier score and a reliability curve (Supplementary Figure S3). Recipient identifiers were not available, so cross validation folds were defined at the biopsy level; performance may be optimistic if repeated biopsies per recipient are present. Group sizes: *N* = 27, *R* = 16. Model evaluation performed using 5-fold stratified cross-validation; ROC-AUC derived from out-of-fold predictions with bootstrap 95% confidence intervals. **(A)** Non zero coefficients retained by the L1 penalized logistic regression model (features ordered by absolute coefficient). **(B)** Receiver operating characteristic curve from 5 fold stratified cross validation using out of fold predicted probabilities; the area under the curve is shown. **(C)** Out of fold predicted probabilities by true label (N versus R). **(D)** Heatmap of z scored expression for the selected genes across biopsies, with samples annotated by phenotype.

A sparse diagnostic signature separates rejection from non rejection in internal evaluation We next built a simple classifier using the top 30 differentially expressed genes as the candidate set. L1 logistic regression reduced the model to eight genes (C4A, CCL19, COPG, UBD, TRB@, DEF6, HLA DRB3, IGHG3) (Figure 7A). In 5 fold stratified cross validation, the out of fold ROC curve gave an AUC of 0.993 (Figure 7B). The predicted probabilities were higher in rejection than in non rejection, with only a few outliers (Figure 7C). The eight genes also showed clear group level shifts on the feature heatmap (Figure 7D). When the full workflow was constrained to the training folds using leakage controlled nested cross validation, discrimination was lower (ROC AUC 0.956; PR AUC 0.943; Supplementary Figure S2). Bootstrap resampling of the out of fold predictions gave a 95% confidence interval of 0.890 to 1.000 for ROC AUC and 0.854 to 1.000 for PR AUC. Calibration was assessed by Brier score (0.128; 95% interval 0.072 to 0.192) and a reliability curve (Supplementary Figure S3). Given the single cohort and the absence of external validation, these performance results are best interpreted as hypothesis generating.

### qPCR profiling demonstrates xenograft-associated immune activation

**Figure 8:**
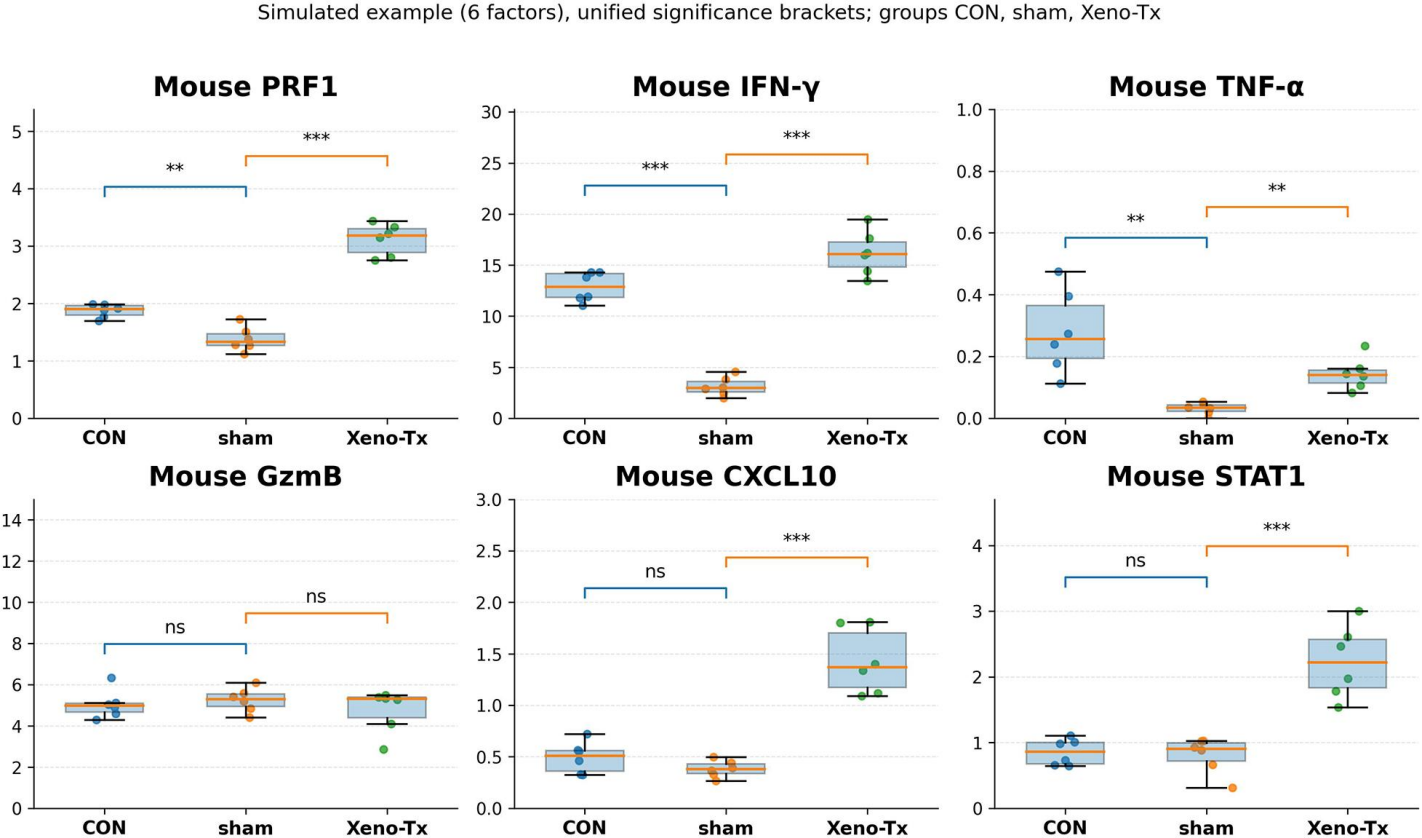
qPCR profiling of inflammatory and cytotoxic markers across CON, sham, and xeno-Tx groups. Expression levels of Prf1, Ifng, Tnf, Gzmb, Cxcl10, and Stat1 are shown for the normal control group (CON), the sham group (right-sided common carotid artery and jugular vein ligation without graft implantation), and the cervical heterotopic cardiac xenotransplantation group (Xeno-Tx; BALB/c donor hearts transplanted into C57 recipients with establishment of graft perfusion). Data are presented as boxplots with overlaid individual data points; boxes indicate the interquartile range and the center line denotes the median. Brackets indicate pairwise comparisons between CON versus sham and sham versus Xeno-Tx. *P* < 0.05, *P* < 0.01, *P* < 0.001.

qPCR results were compared across three groups including CON, sham, and Xeno-Tx. Relative to sham, Xeno-Tx showed higher expression of Ifng, Stat1, Cxcl10, and Prf1, consistent with activation of interferon signaling, chemokine-driven recruitment, and cytotoxic effector programs. Sham showed limited changes compared with CON. Tnf and Gzmb exhibited smaller or no differences across groups at the sampled time point (Figure 8).

## Discussion

Endomyocardial biopsy transcriptomes were analyzed together to show that acute rejection included the upregulation of many different types of immune pathways such as interferon pathways, antigen processing and presenting pathways, chemokine signaling pathways and adaptive receptor pathways (13). Using a complementary approach of looking at markers, the amount of mononuclear and adaptive immune response markers during acute rejection was greater than in non-reject cases. This also agrees with the cellular breakdown of infiltration or activation seen in histologically reviewed cases of acute rejection (14). The internally validated sparse gene signatures developed from this study differentiated biopsy samples for rejections vs. non-rejections with high accuracy. These biomarkers could potentially be used to create a compact panel for generating hypotheses related to acute rejection (15). The enriched interferon and antigen presentation pathways underscore coordinated engagement of innate sensing and adaptive effector mechanisms during rejection (16). Upregulated HLA related genes and accessory molecules (for example HLA DMA, CD74, IFI30) point to heightened antigen processing and presentation capacity within the graft, while interferon regulated transcripts and downstream signaling modules are consistent with an inflammatory microenvironment. The observed enrichment of chemokine pathways and the elevation of CCL19 suggest organized leukocyte recruitment and possible lymphoid like structures within allograft tissue, providing a mechanistic link between transcriptomic signals and immune cell trafficking. From a clinical perspective, transcriptomic profiling has the potential to complement endomyocardial biopsy by providing quantitative and multiplex information that may detect immune activation before overt histologic changes or help resolve borderline findings (17). While this study is not designed to replace existing molecular assays, the convergent signals observed across differential expression, pathway enrichment, and immune scoring support the biological plausibility of the results and motivate external validation. Future work should evaluate whether the proposed sparse signature or expanded pathway derived scores add incremental diagnostic value beyond histology, and whether they track response to intensified immunosuppression or predict longer term outcomes such as cardiac allograft vasculopathy. To evaluate the robustness of the proposed diagnostic signature, feature selection was repeated within the training folds during nested cross-validation. The frequency of gene selection across folds was examined as an indicator of signature stability and reproducibility. Beyond discrimination metrics such as ROC-AUC, translation to clinical diagnostics also requires evaluation of calibration and potential clinical utility. Accordingly, calibration metrics (e.g., Brier score and reliability curves) were examined, and future work may incorporate decision-analytic methods such as decision curve analysis to further assess the clinical usefulness of the model.

The interpretation of immune microenvironment estimates should be cautious because MCP-counter relies on predefined marker genes, and some markers were not represented on the GPL1053 platform. Consequently, several cell populations could not be computed and the inferred immune landscape should be considered indicative rather than definitive. To support biological interpretation, MCP-counter results were interpreted together with pathway enrichment signals such as interferon signaling, antigen presentation, and T-cell receptor pathways. Alternative microarray-compatible deconvolution methods were considered; however, limited probe coverage on this platform may affect their robustness, therefore MCP-counter was retained while explicitly acknowledging these limitations. Beyond confirming immune activation at the transcript level, these findings can be contextualized alongside established molecular tools for rejection surveillance. The Heart Molecular Microscope Diagnostic (MMDx) framework and other targeted gene-expression panels have shown that molecular activity may be present even when histology is graded as no rejection, and can aid in classifying rejection phenotypes and resolving borderline cases (6). In this context, the interferon/antigen-presentation and lymphocyte-recruitment signals observed here are directionally consistent with prior biopsy-based molecular profiling paradigms and support the biological plausibility of the identified pathways and candidate genes.Clinical translation also requires considering how tissue transcriptomics relates to noninvasive surveillance strategies (e.g., peripheral blood gene-expression profiling, donor-derived cell-free DNA, and donor-specific antibodies) (18). Future studies could test whether the proposed sparse gene signature, or pathway- and cell-score summaries, add incremental value beyond routine EMB grading and whether they correlate with contemporaneous blood-based biomarkers, treatment escalation, and short-term recovery of graft function. Longitudinal sampling would be particularly informative to evaluate whether molecular changes precede histologic rejection, track response to intensified immunosuppression, or predict longer-term outcomes such as cardiac allograft vasculopathy. Mechanistically, the enrichment of interferon signaling, antigen processing/presentation, and chemokine pathways suggests coordinated engagement of innate sensing and adaptive effector programs within the allograft. The concurrent elevation of monocytic, B-lineage, and T-cell/cytotoxic signals is consistent with a complex inflammatory microenvironment and supports a multi-compartment view of rejection biology. These observations help prioritize candidate pathways and genes for follow-up studies, including external replication and longitudinal assessments across diverse clinical contexts. Clinical heterogeneity and limited metadata should be considered when interpreting these transcriptomic findings. The GSE2596 dataset was derived from a mixed clinical cohort and although infection-related, Chagas, and other etiologies were excluded to reduce major heterogeneity, detailed clinical covariates such as time after transplantation, immunosuppression regimen, infection status, histologic rejection grade, and the distinction between acute cellular rejection (ACR) and antibody-mediated rejection (AMR) were not available in the public metadata. These factors are known to influence transcriptomic profiles in transplanted organs and therefore may represent potential confounders. Because of this limitation, the molecular signals observed in this study should be interpreted as associations with the rejection phenotype rather than direct causal or mechanistic evidence. Future studies integrating transcriptomic profiling with well-annotated clinical metadata and stratified analyses across rejection subtypes, immunosuppressive regimens, and time post-transplant will be important to refine the biological interpretation of these findings.

The qPCR findings provide additional experimental support for the transcriptomic observations by showing synchronous activation of three key processes, including increased interferon signaling reflected by elevated mRNA levels, chemokine-directed leukocyte recruitment, and cytotoxic effector programs. These changes were observed in a cervical heterotopic cardiac xenotransplantation model in which BALB/c donor hearts were transplanted into the right cervical region of C57 recipients with establishment of graft perfusion. Inclusion of a sham group with right-sided carotid and jugular ligation helps disentangle xenograft-specific immune activation from the effects of surgical and vascular manipulation, strengthening the biological interpretation of the results. The less robust changes observed for Tnf and Gzmb may relate to the sampling time point, tissue compartmentalization, or post-transcriptional regulation, underscoring that not all inflammatory mediators track uniformly at the mRNA level. Future studies incorporating additional time points, paired graft and peripheral sampling, and complementary protein-level and histologic readouts will be important to define pathway dynamics and clarify their relationships with rejection severity and graft function.

There are multiple inherent limitations to the study. First, it is based on a single publically available microarray dataset and represents a retrospective analysis (19). The limited clinical covariates available within the dataset prevented the ability to adjust for other potential confounding factors known to impact transplant outcomes such as immunosuppression regimen, time after transplant, donor-specifc antibodies, and chronic infections that were not included within the dataset's annotations. The sample size was moderate, and although the cross validated AUC was high, this estimate may be optimistic because it was derived from a single patient cohort. Independent validation across multiple centers and platforms will be needed. Additionally, there are several factors which are exampled by the use of probe-based measurements and the mappings of probes to genes which introduces specific biases related to the specific technologies utilized for the study. The emphasis on the necessity for prospective studies is due to the significance of integrating transcriptomics with histological grading, clinical phenotyping, as well as potentially utilizing both RNA sequencing and/or single cell methodologies for refining the mechanisms at the cellular subtype level and extending the translational potential of this work (20). Because feature selection performed on the full dataset can inflate performance estimates, we additionally report leakage-controlled performance using nested cross-validation with within-fold feature selection. Moreover, the public metadata do not include recipient identifiers, so we cannot exclude the possibility of repeated biopsies from the same patient; this may introduce subject-level dependence and inflate cross-validation performance. We have therefore tempered language around model performance, report leakage controlled estimates with confidence intervals and calibration, and emphasize that independent external validation on additional cohorts and platforms is required before any clinical interpretation or deployment.

## Conclusions

Acute cardiac allograft rejection is characterized by coordinated activation of interferon-driven immune programs, antigen presentation, and adaptive immune signaling in EMB transcriptomes (21). A compact gene signature demonstrates strong internal diagnostic performance and provides a foundation for future external validation and translational biomarker development (22). The proposed signature and performance estimates should be viewed as preliminary and hypothesis generating until validated externally.

## Data Availability

The microarray dataset analyzed in this study is publicly available in the Gene Expression Omnibus database under accession number GSE2596 on platform GPL1053 and can be accessed at: https://www.ncbi.nlm.nih.gov/geo/query/acc.cgi?acc=GSE2596. The original data generated from the animal experiments are available from the corresponding author upon reasonable request.

## Supplementary material

Supplementary Figure S1 Missingness pattern after gene level summarization and filtering. (A) Distribution of missing value counts per gene across the 3,968 retained genes (filter required at least 34 of 43 samples). (B) Sample level missingness rates (percent of retained genes missing) across the 43 N/R biopsies.

Supplementary Figure S2 Leakage controlled nested cross validation performance. (A) Receiver operating characteristic curve from nested cross validation using out of fold predictions, where feature selection and model fitting were performed within training folds only (ROC AUC 0.956). (B) Precision recall curve from the same out of fold predictions (PR AUC 0.943).

Supplementary Figure S3 Calibration of leakage controlled nested cross validation predictions. Observed event rates are plotted against mean predicted probabilities across probability bins; the dashed line indicates perfect calibration. Brier score is reported in the panel.

## References

[1] Negargar S Sadeghi S. Early postoperative cardiac complications following heart transplantation: Galen Med J. (2023) 12:e2701. 10.31661/gmj.v12i.2701 37706170 PMC10497256

[2] Rheaume M Aleksova N Madsen JC Benichou G. Chronic rejection series: heart cardiac allograft vasculopathy. Transplantation (2026) 110(1):e28–41. 10.1097/TP.0000000000005453 40653611

[3] McGovern PC Blumberg EA. Differentiation between infection and rejection in the management of cardiac transplant patients. Curr Infect Dis Rep (2001) 3(4):328–32. 10.1007/s11908-001-0069-5 11470022

[4] Goldberg JF Mehta A Bahniwal RK Agbor-Enoh S Shah P. A gentler approach to monitor for heart transplant rejection. Front Cardiovasc Med (2024) 11:1349376. 10.3389/fcvm.2024.1349376 38380175 PMC10876874

[5] Piening BD Dowdell AK Zhang M Loza BL Walls D Gao H Whole transcriptome profiling of prospective endomyocardial biopsies reveals prognostic and diagnostic signatures of cardiac allograft rejection. J Heart Lung Transplant (2022) 41(6):840–8. 10.1016/j.healun.2022.01.1377 35317953 PMC9133065

[6] Halloran PF Madill-Thomsen K Aliabadi-Zuckermann AZ Cadeiras M Crespo-Leiro MG Depasquale EC Many heart transplant biopsies currently diagnosed as no rejection have mild molecular antibody-mediated rejection-related changes. J Heart Lung Transplant (2022) 41(3):334–44. 10.1016/j.healun.2021.08.004 34548198

[7] Chen Y Tso SM Wu F Xu Y Cui L. Profiling shared cytotoxic immune signatures in SLE-associated coronary injury through transcriptomics and machine learning. Immunotargets Ther (2025) 14:1247–66. 10.2147/ITT.S539756 41209049 PMC12595958

[8] Wolf S Melo D Garske KM Pallares LF Lea AJ Ayroles JF. Characterizing the landscape of gene expression variance in humans. PLoS Genet (2023) 19(7):e1010833. 10.1371/journal.pgen.1010833 37410774 PMC10353820

[9] Kaneda T Kurata T Yoshida T Kibata K Yoshioka H Yanagimoto H Massive digital gene expression analysis reveals different predictive profiles for immune checkpoint inhibitor therapy between adenocarcinoma and squamous cell carcinoma of advanced lung cancer. BMC Cancer (2022) 22(1):154. 10.1186/s12885-022-09264-2 35135489 PMC8822674

[10] Chang LY Lee MZ Wu Y Lee WK Ma CL Chang JM Gene set correlation enrichment analysis for interpreting and annotating gene expression profiles. Nucleic Acids Res (2024) 52(3):e17. 10.1093/nar/gkad1187 38096046 PMC10853793

[11] Friedrich M Pohin M Jackson MA Korsunsky I Bullers SJ Rue-Albrecht K IL-1-driven stromal-neutrophil interactions define a subset of patients with inflammatory bowel disease that does not respond to therapies. Nat Med (2021) 27(11):1970–81. 10.1038/s41591-021-01520-5 34675383 PMC8604730

[12] Claros CC Anderson MN Qian W Brockmeier AJ Buckley TA. A machine learning model for post-concussion musculoskeletal injury risk in collegiate athletes. Sports Med (2025) 55(8):1971–82. 10.1007/s40279-025-02196-4 40140234 PMC12460441

[13] Loupy A Duong Van Huyen JP Hidalgo L Reeve J Racapé M Aubert O Gene expression profiling for the identification and classification of antibody-mediated heart rejection. Circulation (2017) 135(10):917–35. 10.1161/CIRCULATIONAHA.116.022907 28148598

[14] Halloran PF Potena L Van Huyen JD Bruneval P Leone O Kim DH Building a tissue-based molecular diagnostic system in heart transplant rejection: the heart molecular microscope diagnostic (MMDx) System. J Heart Lung Transplant (2017) 36(11):1192–200. 10.1016/j.healun.2017.05.029 28662985

[15] Halloran PF Madill-Thomsen KS. The molecular microscope diagnostic system: assessment of rejection and injury in heart transplant biopsies. Transplantation (2023) 107(1):27–44. 10.1097/TP.0000000000004323 36508644

[16] Goldberg JF Truby LK Agbor-Enoh S Jackson AM deFilippi CR Khush KK Selection and interpretation of molecular diagnostics in heart transplantation. Circulation (2023) 148(8):679–94. 10.1161/CIRCULATIONAHA.123.062847 37603604 PMC10449361

[17] Holzhauser L DeFilippis EM Nikolova A Byku M Contreras JP De Marco T The End of endomyocardial biopsy?: a practical guide for noninvasive heart transplant rejection surveillance. JACC Heart Fail (2023) 11(3):263–76. 10.1016/j.jchf.2022.11.002 36682960

[18] Lee DH Usmani A Ravichandran V Wicks T Wu R Wolf-Doty T Relationship between blood and tissue-based rejection-related transcripts in heart transplantation. J Heart Lung Transplant. (2024) 43(3):359–68. 10.1016/j.healun.2023.09.009 37730189

[19] Kobashigawa J Hall S Shah P Fine B Halloran P Jackson AM The evolving use of biomarkers in heart transplantation: consensus of an expert panel. Am J Transplant (2023) 23(6):727–35. 10.1016/j.ajt.2023.02.025 36870390 PMC10387364

[20] Benincasa G Viglietti M Coscioni E Napoli C. “Transplantomics” for predicting allograft rejection: real-life applications and new strategies from network medicine. Hum Immunol. (2023) 84(2):89–97. 10.1016/j.humimm.2022.11.004 36424231

[21] Farcas AO Stoica MC Maier IM Maier AC Sin AI. Heart transplant rejection: from the endomyocardial biopsy to gene expression profiling. Biomedicines. (2024) 12(8):1926. 10.3390/biomedicines12081926 39200392 PMC11351478

[22] Giarraputo A Coutance G Patel JK Fedrigo M Aubert O Varnous S Heart allograft rejection: molecular diagnosis using intra-graft targeted gene expression profiling. Eur Heart J. (2025). 10.1093/eurheartj/ehaf949 41342627

