# Supplementary material for: Transcriptomic signatures and immune microenvironment of acute rejection after heart transplantation: an integrated bioinformatics analysis
Source: Front Cardiovasc Med. 2026 May 25;13:1796145. doi: 10.3389/fcvm.2026.1796145 (PMC13243221; doi:10.3389/fcvm.2026.1796145)
Supplement: Supplementary file 1 [file Datasheet1.pdf]

Supplementary Table S1. Probe-to-gene mapping and identifier clarification for signature genes

Probe ID	Original Annotation	Mapped Gene Symbol	Genomic Locus / Description	Mapping Note
GPL1053_probe_TRB1	TRB@	TRB locus	T-cell receptor beta locus	legacy locus annotation in GPL1053
GPL1053_probe_DEF6	DEF6	DEF6	DEF6 gene	standard HGNC symbol
GPL1053_probe_C4A	C4A	C4A	Complement C4A	standard HGNC symbol
GPL1053_probe_CCL19	CCL19	CCL19	Chemokine ligand 19	standard HGNC symbol
GPL1053_probe_UBD	UBD	UBD	Ubiquitin D	standard HGNC symbol
GPL1053_probe_HLA_DRB3	HLA DRB3	HLA-DRB3	MHC class II gene	HGNC normalized
GPL1053_probe_IGHG3	IGHG3	IGHG3	Immunoglobulin heavy constant gamma 3	HGNC symbol
GPL1053_probe_COPG	COPG	COPG1	Coatomer subunit gamma 1	updated HGNC symbol

Supplementary Table S2. Robustness of differential expression results to alternative preprocessing and inference pipelines

Pipeline	Settings	Genes tested	DEGs (FDR<0.05)	Strict DEGs (FDR<0.05 & |log2FC|>1)	Strict overlap vs base	Top10 pathway overlap
Base	median; >=34/43 (0.78); Gene mean; Welch t-test	3968	1032	135	135 (100.0%)	10
Alt1 mean summary	mean; >=34/43 (0.78); Gene mean; Welch t-test	3968	1033	135	135 (100.0%)	10
Alt2 stricter missing	median; >=39/43 (0.90); Gene mean; Welch t-test	2974	840	106	106 (78.5%)	6
Alt3 looser missing	median; >=31/43 (0.70); Gene mean; Welch t-test	4358	1079	146	135 (100.0%)	9
Alt4 no imputation	median; >=34/43	3968	1031	159	135 (100.0%)	8

	(0.78); None; Welch t-test					
Alt5 Mann Whitney	median; >=34/43 (0.78); Gene mean; Mann–Whitney U	3968	1044	135	135 (100.0%)	10
