# Supplementary figures and images for: Transcriptomic signatures and immune microenvironment of acute rejection after heart transplantation: an integrated bioinformatics analysis
Source: Front Cardiovasc Med. 2026 May 25;13:1796145. doi: 10.3389/fcvm.2026.1796145 (PMC13243221; doi:10.3389/fcvm.2026.1796145)

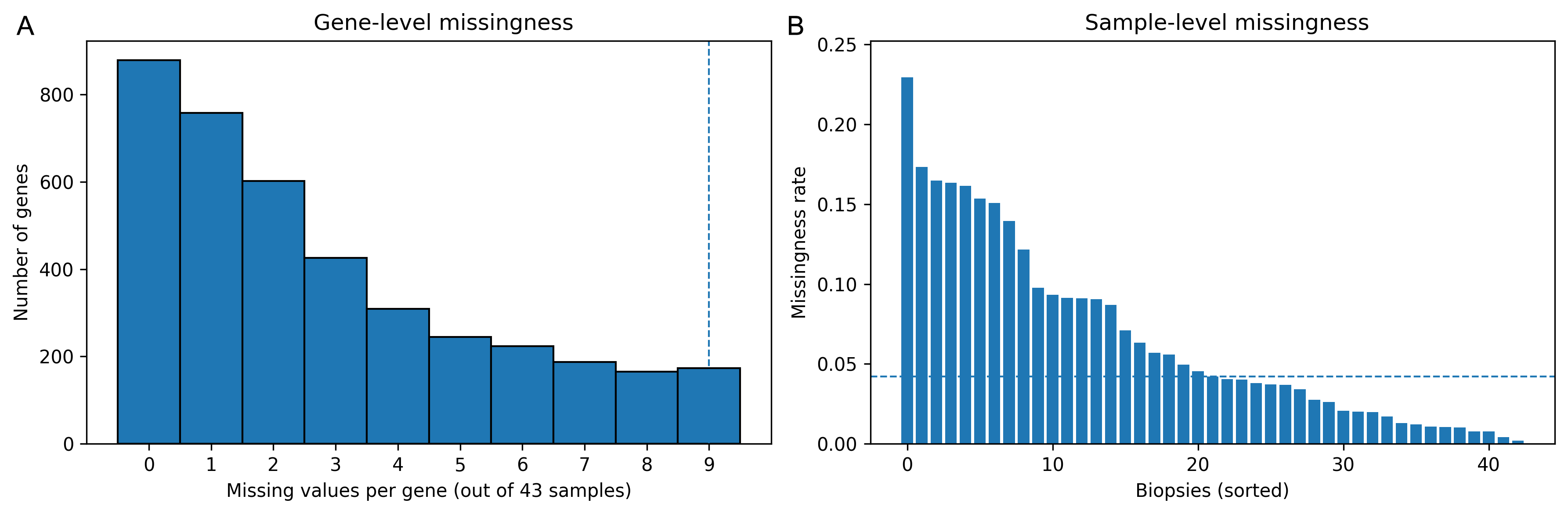

Supplement: Supplementary Figure S1 — Missingness pattern after gene level summarization and filtering. (A) Distribution of missing value counts per gene across the 3,968 retained genes (filter required at least 34 of 43 samples). (B) Sample level missingness rates (percent of retained genes missing) across the 43 N/R biopsies. [file Image1.tiff]

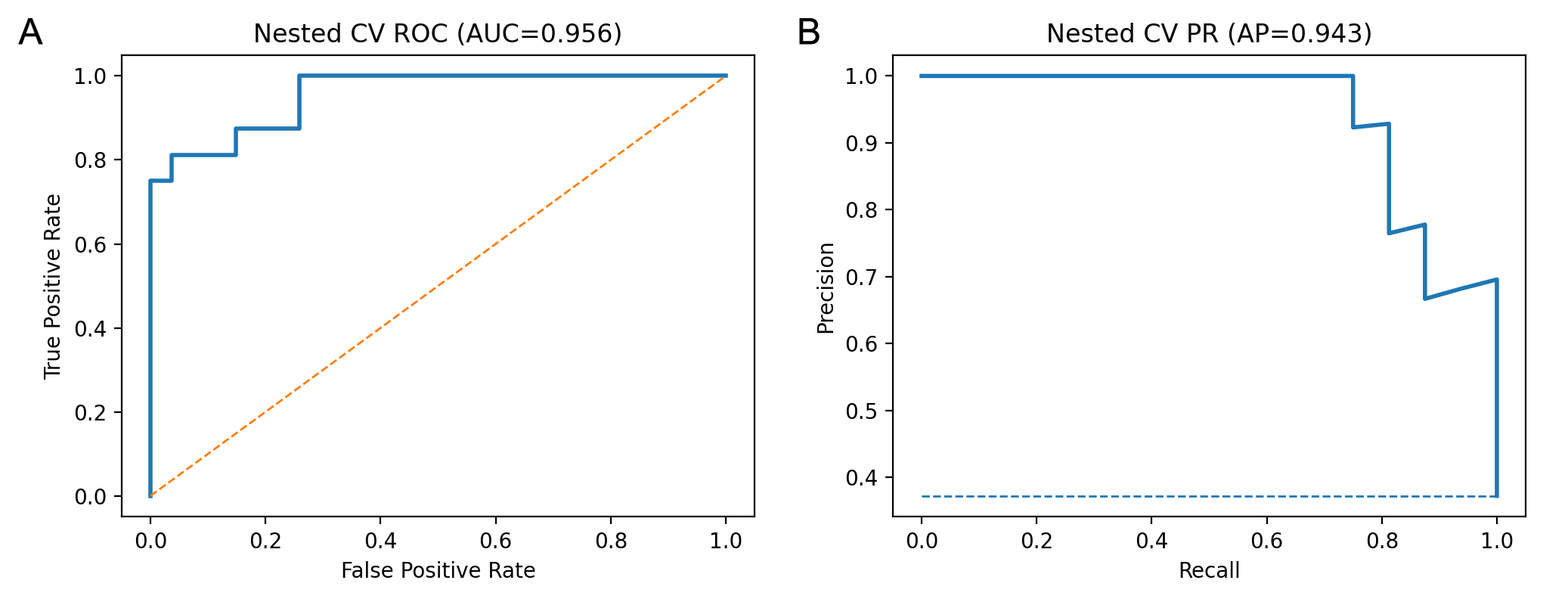

Supplement: Supplementary Figure S2 — Leakage controlled nested cross validation performance. (A) Receiver operating characteristic curve from nested cross validation using out of fold predictions, where feature selection and model fitting were performed within training folds only (ROC AUC 0.956). (B) Precision recall curve from the same out of fold predictions (PR AUC 0.943). [file Image2.tiff]

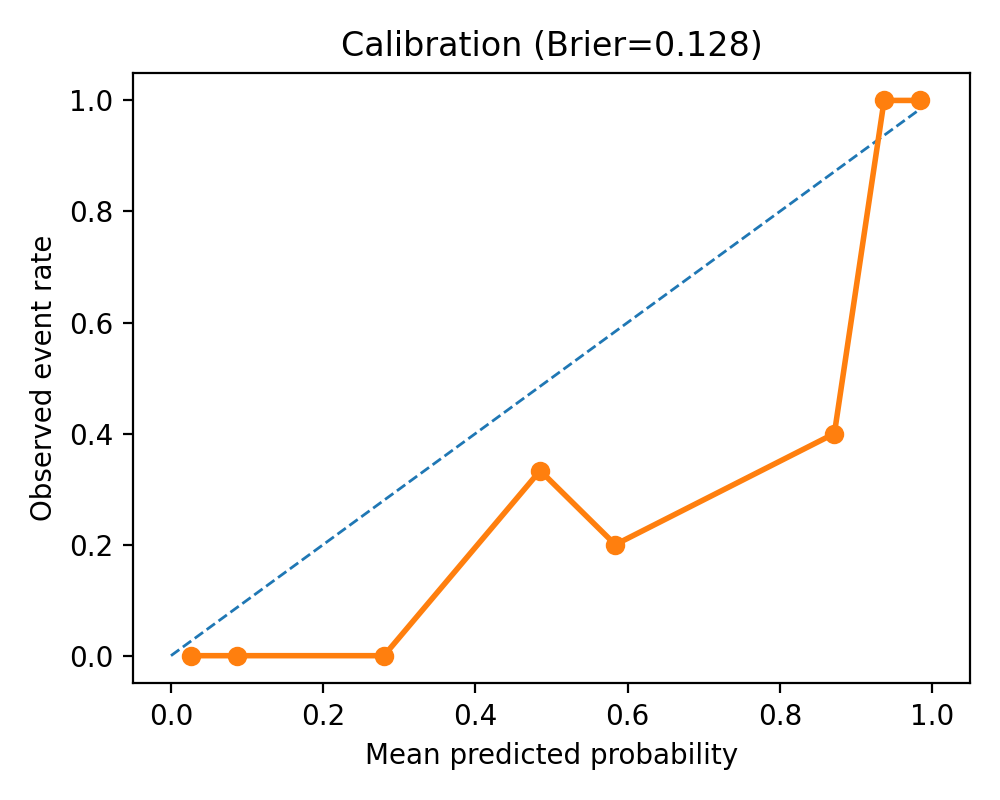

Supplement: Supplementary Figure S3 — Calibration of leakage controlled nested cross validation predictions. Observed event rates are plotted against mean predicted probabilities across probability bins; the dashed line indicates perfect calibration. Brier score is reported in the panel. [file Image3.tiff]
